# Carprofen inhibits the release of matrix metalloproteinases 1, 3, and 13 in the secretome of an explant model of articular cartilage stimulated with interleukin 1β

**DOI:** 10.1186/ar4424

**Published:** 2013-12-30

**Authors:** Adam Williams, Julia R Smith, David Allaway, Pat Harris, Susan Liddell, Ali Mobasheri

**Affiliations:** 1 Musculoskeletal Research Group, School of Veterinary Medicine and Science, Faculty of Medicine and Health Sciences, The University of Nottingham, Sutton Bonington Campus, Sutton Bonington LE12 5RD, United Kingdom; 2 Bruker UK Limited, Coventry CV4 9GH, United Kingdom; 3 WALTHAM® Centre for Pet Nutrition, Waltham-on-the-Wolds, Melton Mowbray, Leicestershire LE14 4RT, United Kingdom; 4 Proteomics Laboratory, School of Biosciences, The University of Nottingham, Sutton Bonington Campus, Leicestershire LE12 5RD, United Kingdom; 5 The D-BOARD European Consortium for Biomarker Discovery, The University of Nottingham, University Park, Nottingham NG7 2RD, UK; 6 Arthritis Research UK Centre for Sport, Exercise and Osteoarthritis, Nottingham University Hospitals, Nottingham NG7 2UH, United Kingdom; 7 Arthritis Research UK Pain Centre, The University of Nottingham, Queen’s Medical Centre, Nottingham NG7 2UH, United Kingdom; 8 Medical Research Council and Arthritis Research UK Centre for Musculoskeletal Ageing Research, The University of Nottingham, Queen’s Medical Centre, Nottingham NG7 2UH, United Kingdom; 9 Center of Excellence in Genomic Medicine Research (CEGMR), King Fahd Medical Research Center (KFMRC), King AbdulAziz University, Jeddah 21589, Kingdom of Saudi Arabia; 10 Schools of Life Sciences and Pharmacy, University of Bradford, Richmond Road, Bradford BD7 1DP, United Kingdom; 11 Faculty of Medicine and Health Sciences, The University of Nottingham, Sutton Bonington Campus, Sutton Bonington LE12 5RD, United Kingdom

## Abstract

**Introduction:**

Arthritic diseases are characterized by the degradation of collagenous and noncollagenous extracellular matrix (ECM) components in articular cartilage. The increased expression and activity of matrix metalloproteinases (MMPs) is partly responsible for cartilage degradation. This study used proteomics to identify inflammatory proteins and catabolic enzymes released in a serum-free explant model of articular cartilage stimulated with the pro-inflammatory cytokine interleukin 1β (IL-1β). Western blotting was used to quantify the release of selected proteins in the presence or absence of the cyclooxygenase-2 specific nonsteroidal pro-inflammatory drug carprofen.

**Methods:**

Cartilage explant cultures were established by using metacarpophalangeal joints from horses euthanized for purposes other than research. Samples were treated as follows: no treatment (control), IL-1β (10 ng/ml), carprofen (100 μg/ml), and carprofen (100 μg/ml) + IL-1β (10 ng/ml). Explants were incubated (37°C, 5% CO_2_) over twelve day time courses. High-throughput nano liquid chromatography/mass spectrometry/mass spectrometry uncovered candidate proteins for quantitative western blot analysis. Proteoglycan loss was assessed by using the dimethylmethylene blue (DMMB) assay, which measures the release of sulfated glycosaminoglycans (GAGs).

**Results:**

Mass spectrometry identified MMP-1, -3, -13, and the ECM constituents thrombospondin-1 (TSP-1) and fibronectin-1 (FN1). IL-1β stimulation increased the release of all three MMPs. IL-1β also stimulated the fragmentation of FN1 and increased chondrocyte cell death (as assessed by β-actin release). Addition of carprofen significantly decreased MMP release and the appearance of a 60 kDa fragment of FN1 without causing any detectable cytotoxicity to chondrocytes. DMMB assays suggested that carprofen initially inhibited IL-1β-induced GAG release, but this effect was transient. Overall, during the two time courses, GAG release was 58.67% ± 10.91% (SD) for IL-1β versus 52.91% ± 9.35% (SD) with carprofen + IL-1β.

**Conclusions:**

Carprofen exhibits beneficial anti-inflammatory and anti-catabolic effects *in vitro* without causing any detectable cytotoxicity. Combining proteomics with this explant model provides a sensitive screening system for anti-inflammatory compounds.

## Introduction

Articular cartilage is a highly specialized load-bearing tissue that covers the ends of long bones in synovial joints and provides a strong and resilient surface for smooth and frictionless articulation as well as cushioning of the underlying bone [1,2]. The major biologic constituents of the extracellular matrix (ECM) of cartilage include collagens, proteoglycans, and noncollagenous proteins [1]. The chondrocyte is the main cell type found within the ECM of skeletally mature cartilage [3]. Chondrocytes synthesize all the ECM components in cartilage [3] and maintain this macromolecular framework in response to biochemical and biomechanical stimuli [4]. The ECM contains a specific combination of structural proteins and glycoproteins that are unique to cartilage. In addition, the ECM contains a number of other smaller noncollagenous proteins, including thrombospondin 1 (TSP-1) and fibronectin 1 (FN1). Fragments of FN1 are released in osteoarthritis (OA) and are thought to promote further cartilage degradation by upregulating catabolic signaling [5,6].

In diseases such as OA and rheumatoid arthritis (RA) chondrocytes are targeted, via specific cell-surface cytokine receptors, by pro-inflammatory cytokines such as interleukin-1β (IL-1β) and tumour necrosis factor-α (TNF-α). Although other pro-inflammatory cytokines (i.e. IL-6, IL-8 and IL-17) are involved, IL-1β and TNF-α are the predominant pro-inflammatory and catabolic cytokines involved in joint disease initiation and progression [7,8]. These pro-inflammatory cytokines suppress collagen and proteoglycan synthesis and drive inflammatory signaling, and protease expression/activation [9].

**Figure 1:**
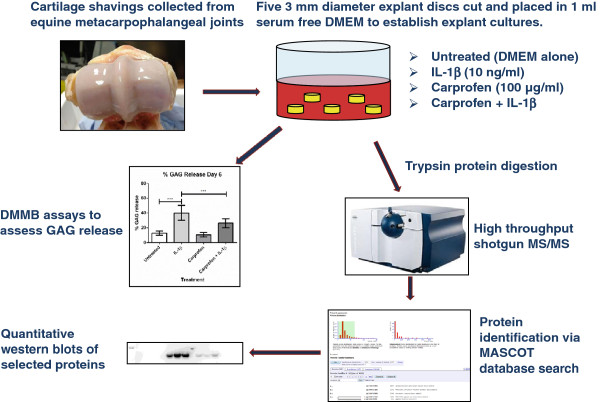
**Schematic overview of the experimental design used in this study.** Explant cultures were incubated for 6 days in designated treatments before conditioned supernatant was removed and treatment replenished for cultures up to 12 days.

Previous studies from our research group have used an explant model of articular cartilage to study the major proteins released in response to IL-1β stimulation [10]. Subsequent high-throughput MS analysis of this model identified qualitative differences in MMP-1, -3, and -13 expression between untreated and IL-1β-stimulated explants in the spent culture media [11]. In this study, we used high-throughput proteomics and quantitative western blotting to evaluate the release of these MMPs in response to IL-1β stimulation in the presence and absence of carprofen (marketed as Rimadyl) [12], a nonsteroidal anti-inflammatory drug (NSAID) developed by Pfizer Animal Health. Carprofen is a selective cyclooxygenase 2 (COX-2) inhibitor capable of blocking synthesis of the key inflammatory bioactive lipids like prostaglandin E_2_ (PGE_2_). It is used clinically to provide 24-hour relief of pain and inflammation in geriatric dogs and horses with joint pain, OA, hip dysplasia, and other forms of joint disease. The principal hypothesis of this study was that by combining proteomics with western blotting, we could determine and characterize effects of anti-inflammatory compounds (by using carprofen as an anti-inflammatory agent) in an *in vitro* model of cartilage (Figure 1). MMP-1, -3, and -13 were studied as surrogate *in vitro* biomarkers of inflammation to determine whether carprofen has the capacity to reduce the release of these catabolic enzymes. The effect of carprofen on cytokine-stimulated GAG release was also studied in cultures up to 6 and 12 days.

## Methods

### Animal tissues and statement of ethical approval

The animals used in this study were sourced from two UK-based abattoirs. Animals were euthanized for nonresearch purposes, having been stunned before slaughter in accordance with Welfare of Animals (Slaughter or Killing) Regulations 1995 [13]. Approval for the use of abattoir-derived animal tissues was obtained from the local Ethical Review Committee (Ethics Committee of the School of Veterinary Science and Medicine) with input from members of the University of Nottingham Animal Welfare and Ethical Review Body (AWERB).

### Cartilage dissection and explant culture

Macroscopically normal articular cartilage samples were obtained from weight-bearing regions of the metacarpophalangeal joints of horses of mixed breed, age, and sex. Articular cartilage was harvested from the metacarpophalangeal joints. The joints were first shaved to remove excess hair and disinfected by washing with Hibiscrub and then Trigene, before being soaked in bleach for 1 hour. Afterward, the skin and flexor tendon was carefully removed from the joint without breaking into the synovial structure. A sterile scalpel was used to open the joint, which was then washed with sterile PBS (Gibco). Articular cartilage shavings of equal thickness were taken from the end surface of the third metacarpal and placed in 50 ml of serum-free DMEM (Thermo Scientific) + 4% Pen/Strep (Sigma) (Penicillin/Streptomycin solution: (5,000 units/ml penicillin, 5 mg/ml streptomycin)) (pre-warmed to 37°C). These cartilage shavings were transferred to a Falcon tube containing washing solution (sterile PBS + 10% Pen/Strep) and were twice washed with rotation for 20 minutes.

Cartilage-explant discs were prepared by using a 3-mm biopsy punch (Kai Medical), with discs placed into wells (five discs per 1 ml DMEM + 2% Pen/Strep) before incubation for 24 hours (37°C, 5% CO_2_). After this equilibration period, media was removed and replaced with treatment media required for the purposes of the experiment. Once explants had been treated and incubated for the desired 6-day time courses, the supernatants and cartilage explants were placed in labeled Eppendorf tubes and stored at −80°C. Protease inhibitors (Sigma) were added to the supernatant samples at the time of removal and storage.

### Time course

After dissection and explant culture protocols were followed, wells (five explants discs with 1 ml culture media) were set up for each of the following treatments: untreated culture media (control), IL-1β (10 ng/ml), carprofen (100 μg/ml), or carprofen (100 μg/ml) + IL-1β (10 ng/ml). Explants were incubated (37°C, 5% CO_2_) for 6 days before removal and storage of supernatants and the addition of fresh treatment media. This process was repeated to provide a time course with two assay points: 0 to 6 days and 6 to 12 days. This experimental set-up was completed with cartilage from three animals, with three replicates of each treatment from each animal.

### NanoLC-MS/MS

MS analysis was completed on four culture supernatants after 6 days of incubation: two from one animal (untreated control and IL-1β stimulated) and two from a pool of supernatants from three animals (untreated control and IL-1β stimulated). To prepare samples for MS, reduction of disulfide bonds was performed by addition of DTT (Bio-Rad) to a final concentration of 10 m*M*, followed by vortexing and incubation at 37°C for 30 minutes. Alkylation of thiol groups performed by addition of IAA (Bio-Rad) to a 55 m*M* concentration, vortexed and incubated for 45 minutes at 37°C (in the dark). Ice-cold acetone (1.2 ml) (Fisher) was added and incubated at −20°C for 1 hour, and then after centrifugation at 15,000 rcf for 5 minutes, the acetone was discarded. Proteins in the pellet were digested with 20 ng/μl trypsin gold (Promega) at 37°C overnight (16 hours). Trypsin digestion was terminated by addition of formic acid at a final concentration of 0.1%. Samples were zip-tipped with C18 resin (Millipore) by using 20 μl 50% methanol and 0.1% formic acid to elute. Excess solvent was evaporated off by heating at 70°C, to leave 10 μl of samples, which was transferred to glass vials ready to be loaded onto the nanoLC column. For each sample, 5 μl was loaded and analyzed by nanoLC-MS/MS on an amaZon ETD (Bruker). A flow rate of 250 nl/min was used to separate peptides. Solution A (100% H_2_O + 0.1% formic acid) and solution B (100% ACN + 0.1% formic acid) were set to create a gradient of 10% solution B to 30% solution B over the course of an hour (0.3% per minute). From each MS scan, the five most abundant peptides were selected for fragmentation.

### MS data processing

Mascot Daemon was used to search the Swiss-Prot database (all mammals) by submitting the .MGF files. The search parameters were as follows: Instrument: ESI-TRAP, peptide charge, 2+ and 3+ ions; peptide tolerance, 0.5 Da; ^13^C = 1; max missed cleavages = 1. Fixed modifications: carbamidomethyl (C) and variable modifications: oxidation (M). Individual ions Mascot scores above 42 indicated identity or extensive homology. Only protein identifications with probability-based protein family mascot MOWSE scores above the significant threshold of >42 (*P <* 0.05) were accepted and included if consistently identified in the specific treatments.

### SDS-PAGE with Protean 3 mini-gels

For each sample, 10 μl collected culture supernatant was added to 10 μl of Laemmli sample buffer (62.5 m*M* Tris–HCl, pH 6.8, 2% SDS, 25% glycerol, 0.01% wt/vol bromophenol blue) (Bio-Rad) containing dithiothreitol (DTT) (Bio-Rad) 0.15 *M*). Samples were heated for 5 minutes at 95°C before loading into a 15-well 10% acrylamide gel. To reference protein molecular weights Precision Plus Protein Standards (Bio-Rad) were also loaded. A voltage of 120 V was applied until the dye front reached the bottom of the gel.

### Western blotting

Western blots on days 0 to 6 of incubation were completed in three animals. Each sample of 50 μl was concentrated by lyophilization (Heraeus-Christ) before resuspension in 20 μl Laemmli buffer (Bio-Rad) before performing SDS-PAGE (see previous method). Positive controls were prepared by crushing articular cartilage with a mortar and pestle under liquid nitrogen, and susended in Laemmli buffer. After SDS-PAGE, gels were electroblotted onto low-fluorescence PVDF membrane (GE Healthcare) at 80 V for 2 hours. Membranes were blocked for 1 hour with 5% BSA (Sigma) in PBS-Tween 20 (0.1%) (Fisher). Primary antibodies were diluted as required in blocking solution before overnight incubation at 4°C. Dilutions for primary antibodies were as follows: MMP-1 (1:800 dilution, Aviva), MMP-3 (1:1,000 dilution, Aviva), MMP-13 (1:1,000 dilution, Aviva), FN1 (1:1,000 dilution, Aviva), TSP1 (1:1,000, Abcam). The antibodies from Aviva systems biology were specifically designed to cross-react with multiple different species, including the horse.

After the overnight primary incubation, membranes were incubated with secondary antibodies for 1 hour. The secondary antibodies used in this study were all HRP conjugated and diluted to appropriate working concentrations by using 3% BSA blocking solution. Secondary antibodies were either anti-rabbit (1:100,000 dilution, Bio-Rad) for MMP-1, MMP-3, MMP-3, and FN1 primary antibodies or anti-rabbit/mouse (1:40,000 dilution, Dako) for the TSP-1 primary antibody.

Ampliflu Red Western Blot Kit (Sigma) was used to stain protein bands with red fluorescence, which was next detected on an FX-Imager (Bio-Rad) by using Quantity One software (Bio-Rad). Densitometry was performed on the images obtained by using ImageJ (NIH). Statistical analysis applying an unpaired *t*-test was completed, and the results graphically displayed using GraphPad Prism 6.

Chondrocyte cell death in the cartilage explants was monitored indirectly by using western blotting to detect β-actin release by explant cultures as an indicator of cytotoxicity and cell lysis. We compared the release of β-actin in untreated (control) cartilage explants and those that were stimulated with IL-1β, carprofen, and carprofen + IL-1β. For this purpose, we used a primary antibody to β-actin (1:7,000 dilution, Sigma) and a secondary anti-mouse HRP conjugate (1:100,000 dilution, Cell Signaling).

### DMMB assays

For evaluation of proteoglycan release, the metachromatic dye 1,9 dimethylmethylene blue (DMMB) was used to quantify the amount of sulfated glycosaminoglycans (GAGs) released into the medium. DMMB, papain, and chondroitin sulfate standards (Sigma) were prepared according to the method developed by Farndale *et al.*[14]. Supernatant samples were diluted to appropriate concentrations, to be within the accurate range (0 to 75 μg/ml) of the standard curve. On a 96-well plate, 40 μl of blank papain solution (duplicate), each standard (duplicate) and sample dilutions (triplicate) were added. Next, 200 μl of DMMB solution was added to each well, before reading the plate at 540 nm (spectrophotometer) within 10 minutes. To calculate the percentage of total GAG release, corresponding explant discs were digested overnight by incubating with papain solution at 60°C, before their GAG content was measured, also by DMMB assay. Total GAG release levels per well were calculated, before dividing the total GAG release by measurements for individual supernatant samples to provide the percentage GAG release in response to various treatments. Measurements from three animals (three replicates per treatment) were combined to provide final values for each incubation period.

GraphPad Prism 6 software was used produce graphic images and complete statistical analysis with one-way ANOVA by using the Tukey multiple comparison test.

## Results and discussion

### Carprofen decreases IL-1β-stimulated MMP-1 release

**Figure 2:**
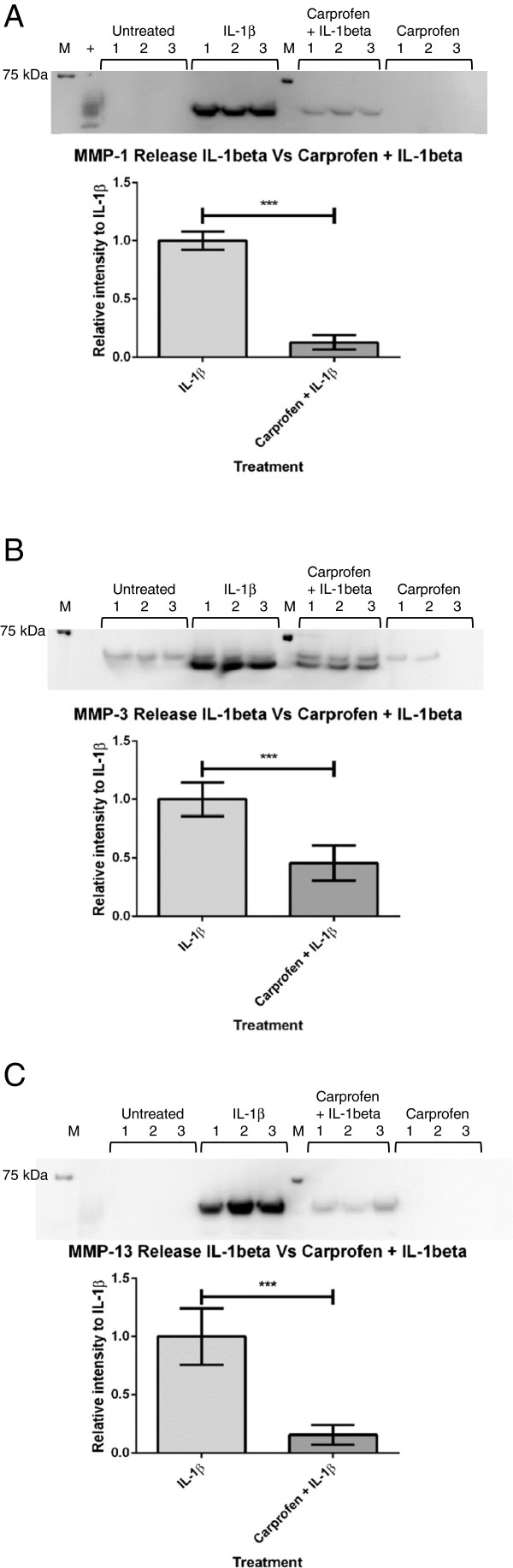
**Western blotting of MMP-1, -3, and -13 release in the secretome of cartilage explants in culture. (A)** Representative MMP-1 western blot image and densitometric analysis showing significant decrease in IL-1β-stimulated release of MMP-1 in response to carprofen treatment. **(B)** Representative MMP-3 western blot image and densitometric analysis displaying the significant reduction of IL-1β MMP-3 release attributed to carprofen treatment. **(C)** Representative MMP-13 western blot image and densitometric analysis showing significantly higher MMP-13 release in the presence of IL-1β alone, compared with carprofen + IL-1β. All cartilage-explant supernatants were incubated for 6 days. This experimental design was carried out with tissues obtained from three separate animals, with three treatment replicates from each animal (*n =* 9). Statistical analysis completed = unpaired *t*-test. Treatments were as follows: untreated control, IL-1β (10 ng/ml), carprofen (100 μg/ml), or carprofen (100 μg/ml) + IL-1β (10 ng/ml). Error bars indicate standard deviation. ***P < 0.001. M, molecular weight markers; +positive control.

Mass spectrometric analysis of explant cultures incubated for 6 days identified MMP-1 in IL-1β-stimulated samples analyzed from all the animals (Table 1). MMP-1 was not identified by MS in untreated control samples. Untreated samples and carprofen-only treatments did not produce detected levels of MMP-1 by using western blot analysis. Western blots of day-6 explant cultures treated with IL-1β and IL-1β + carprofen contained protein bands at around 53 kDa, consistent with the predicted molecular mass of MMP-1 (Figure 2A). Densitometry and statistical analysis showed a significant decrease in IL-1β-stimulated MMP-1 release in response to carprofen treatment.

**Table 1:** Summary of proteins identified in untreated control and IL-1β-stimulated samples with qualitative nanoLC-MS/MS

**Protein**	**Accession number**	**Mascot scores**			
		**Individual animal Untreated control**	**Pooled untreated control**	**Individual animal IL-1β stimulated**	**Pooled IL-1β stimulated**
**ECM proteins**					
Cartilage oligomeric matrix protein	COMP_BOVIN	4090	4763	7555	10590
Fibronectin	FINC_BOVIN	4422	6222	3786	3492
Chondroadherin	CHAD_HUMAN	1022	3069	1978	3689
Thrombospondin 1	TSP1_BOVIN	741	1256	3456	2782
Aggrecan core protein	PGCA_HUMAN	1815	3389	2258	6412
Decorin	PGS2_HORSE	665	802	513	1130
Biglycan	PGS1_HORSE	1099	1062	2047	1720
Fibromodulin	FMOD_HUMAN	1307	3200	1381	1930
Collagen alpha-1(II) chain	CO2A1_BOVIN	137	59	133	82
Collagen alpha-2(VI) chain	CO6A2_HUMAN	190	79		
Collagen alpha-1(X) chain	COAA1_MOUSE	77	102		
Prolargin	PRELP_MOUSE	124	147	311	301
Cartilage intermediate-layer protein 1 (CILP-1)	CILP1_MOUSE	201	789	423	264
Proteoglycan 4 (Lubricin)	PRG4_HUMAN	139	49		59
SPARC (secreted protein acidic, cysteine rich)	SPRC_PIG	108	96		
**Non-ECM secreted proteins**					
Clusterin	CLUS_HORSE	2767	4723	1785	4876
Chitinase-3-like protein 1 (YKL-40)	CH3L1_BOVIN	574	1178	414	2018
Serum amyloid A protein	SAA_CANFA	43			
	SAA_HORSE		55	219	436
Metalloproteinase inhibitor 1	TIMP1_HORSE	83	312	457	146
Alpha-1-antiproteinase 2	A1AT2_HORSE	99	342	50	
MMP-1	MMP1_HORSE			1678	1586
MMP-3	MMP3_HORSE	653	956	5441	4580
MMP-13	MMP13_HORSE			1700	699
TNF-R superfamily, member 11b (Osteoprotegerin)	TR11B_RAT	252	319	388	638
Procollagen C-endopeptidase enhancer 2	PCOC2_HUMAN	163	171		
Lysozyme C	LYSM_BOVIN	72	168	179	148
Secreted frizzled-related protein 3	SFRP3_BOVIN	74	98		
Lactadherin	MFGM_PIG	83	63		54
Extracellular superoxide dismutase [Cu-Zn]	SODE_HUMAN	67	72		
Macrophage migration inhibitory factor	MIF_HUMAN			91	86
Ribonuclease 4	RNAS4_HUMAN		48	46	59
**Intracellular proteins**					
Vimentin	VIME_PANTR		84		
	VIME_PIG			2361	3837
Alpha-enolase	ENOA_BOVIN			506	686
Phosphoglycerate mutase 1	PGAM1_MOUSE			149	104
Phosphatidylethanolamine-binding protein 1	PEBP1_HUMAN			423	148
Phosphoglycerate kinase 1	PGK1_HORSE			792	651
Pyruvate kinase isoforms M1/M2	KPYM_HUMAN			488	245
Purine nucleoside phosphorylase	PNPH_HUMAN			175	134
Glyceraldehyde 3-phosphate dehydrogenase	G3P_PIG			208	115
Thioredoxin	THIO_HORSE			145	143
**Other**					
Serum albumin	ALBU_BOVIN			283	86
Pancreatic trypsin inhibitor	BPT1_BOVIN	283	477	281	703
Trypsin	TRYP_PIG	111		106	746
Keratin, type II cytoskeletal 3	K2C3_RABIT	151	277	110	

MMPs (-1, -3, and -13) were identified from this high-throughput proteomic analysis and investigated further in this study. Identifications were attained by using Mascot Daemon to search the Swiss-Prot database (All mammals). Only peptide identifications with a probability-based protein family mascot MOWSE score above the significant threshold of >42 (with a statistical significance level of *P* < 0.05) were included. MMPs (-1, -3, and -13) were identified from this high-throughput proteomic analysis and investigated further in this study.

### Carprofen decreases IL-1β-stimulated MMP-3 release, whereas TSP-1 is unaffected

MMP-3 was identified in both untreated and IL-1β-stimulated explant media (after 6 days of incubation) by qualitative MS analysis (Table 1). The mascot scores were considerably higher in IL-1β treatments compared with untreated samples, giving an indication that IL-1β stimulation was having an impact on MMP-3 release. Because the MS analysis was qualitative, western blotting was used to provide accurate quantification of MMP-3 release.

Western blotting was used to quantify changes in MMP-3 release from explants in response to IL-1β stimulation and/or carprofen treatment. Upregulation of MMP-3 and its increased proteolytic activity is known to target several ECM substrates [15]. Western blotting for TSP-1 was therefore carried out by using the same membranes to determine whether the release of this ECM protein is affected by IL-1β stimulation and carprofen treatment. Bands for MMP-3 were visible at different intensities at 54 kDa in all treatment groups. An additional band a few kDa below 54 kDa appeared in all samples stimulated with IL-1β. This lower band corresponds to the active form of MMP-3 from which the propeptide domain has been cleaved.

Western blots of untreated and carprofen-alone samples revealed only a single band at 54 kDa corresponding to the nonactive zymogen form of MMP-3 [16,17].

Comparisons of MMP-3 release in explant cultures 6-day incubations (*n* = 3) (Figure 2) provided evidence to support the assertion that carprofen significantly decreases cytokine-stimulated MMP-3 release. MMP-mediated degradation of the ECM may be inhibited by carprofen, as treatment with this NSAID reduced the IL-1β-stimulated release and activation of MMP-3.

TSP-1 blots produced expected bands around 125 kDa that were visible in only IL-1β- and carprofen + IL-1β-stimulated samples. Densitometry did not show any significant differences in the presence of carprofen alone (See Additional file 1: Figure S1).

### Carprofen decreases IL-1β-stimulated MMP-13 release

Release of MMP-13 was not detected by MS analysis of untreated explant culture media. However, MMP-13 was found in the media from all explant cultures stimulated with IL-1β (Table 1). This suggests that MMP-13 release was initiated in the explant model after IL-1β stimulation, but western blotting was required for quantification.

MMP-13 release was increased by IL-1β stimulation, and corresponding bands at 54 kDa were seen only in the presence of this cytokine (that is, not in untreated or carprofen-only explant cultures) (Figure 2). For IL-1β + carprofen treatments, faint bands were present after 6 days of incubation. Significantly less MMP-13 release occurred in the presence of carprofen when the IL-1β and IL-1β + carprofen samples were compared.

### Carprofen treatment decreases the IL-1β-stimulated production of the 60 kDa fragment of FN1

**Figure 3:**
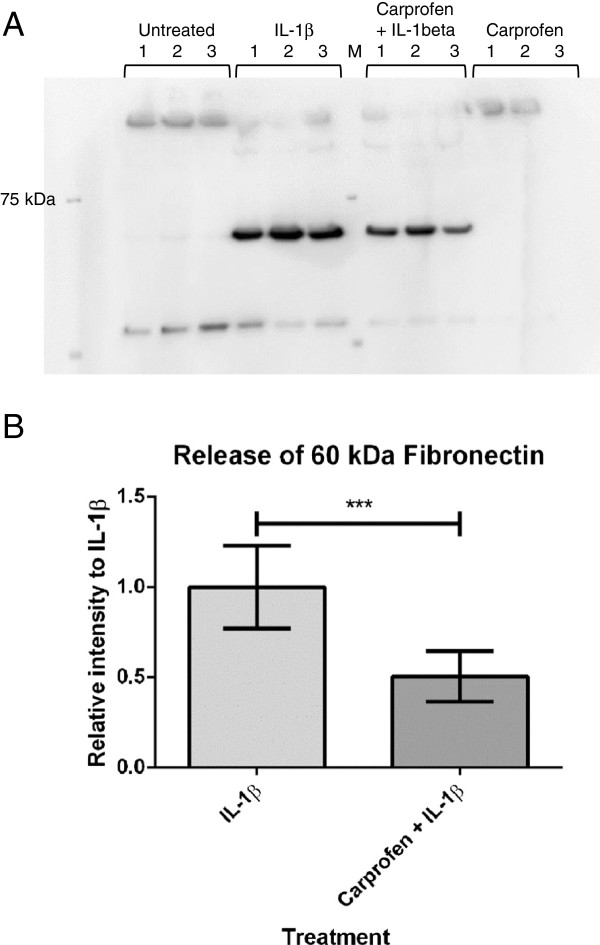
**IL-1β-stimulated degradation of FN1 and the production of its 60 kDa fragment is significantly reduced after carprofen + IL-1β treatment. (A)** Representative FN western blot image. The band at about 230 kDa represents the glycosylated FN monomer. The 60 kDa band fragment was released when explants were stimulated with IL-1β. **(B)** Graphic representation of densitometry for FN. Explant cultures were completed for 6 days in three separate animals, with three treatment replicates on each occasion (*n = 9*). Explant treatments: untreated control, IL-1β (10 ng/ml), carprofen (100 μg/ml), or carprofen (100 μg/ml) + IL-1β (10 ng/ml). Error bars indicate standard deviation. An unpaired *t*-test was applied to assess significance. ***P < 0.001.

Western blotting of FN revealed bands at 230 kDa representing the glycosylated monomer of this proteoglycan, and a further band at 60 kDa (Figure 3). Stimulation with IL-1β increased the density of the lower band (60 kDa), suggesting that this is a degradation product induced by this pro-inflammatory cytokine. A significant reduction was noted in the intensity of this band when carprofen was present along with the IL-1β.

### Carprofen does not inhibit GAG release throughout the entire time course

**Figure 4:**
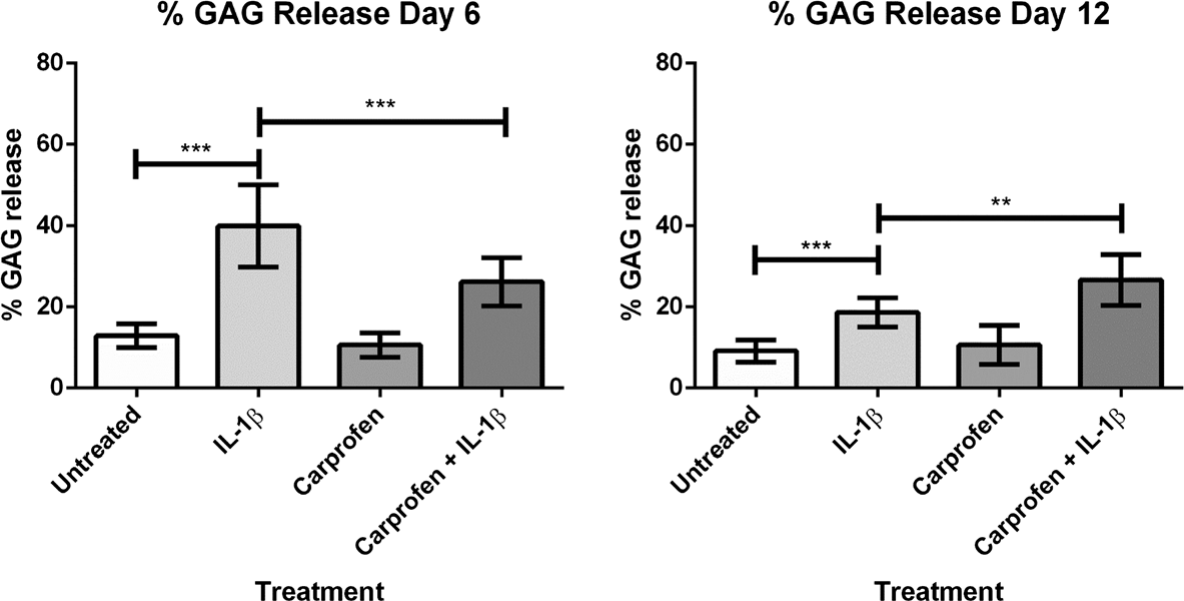
**Percentage GAG release throughout the time course with IL-1β stimulation and carprofen treatment.** The following treatments were applied: untreated control, IL-1β (10 ng/ml), carprofen (100 μg/ml) + IL-1β (10 ng/ml), or carprofen (100 μg/ml). The percentage GAG release was measured after 0 to 6 days and 6 to 12 days of incubation. The day 6 GAG-release data confirmed that carprofen slows IL-1β-stimulated GAG release. The GAG-release measurements for the day 12 time point would have been influenced by GAG loss during the earlier culture period. Carprofen + IL-1β samples showed higher GAG release at day 12 than did IL-1β alone, because of the high loses that had already occurred during days 0 to 6. Explant culture time courses were completed by using tissues from three separate animals, with three treatment replicates for each experiment. **P < 0. 01; ***P < 0. 001. Error bars indicate standard deviation.

A significant increase in GAG release in IL-1β-stimulated cultures was found compared with untreated controls (Figure 4). Carprofen treatment alone did not have any effect on the level of GAG release, as GAG levels were similar to untreated explant cultures throughout the entire time course. During 0 to 6 days of culture, comparing IL-1β with carprofen + IL-1β shows that carprofen caused a significant decrease in GAG release. The later time point of 6 to 12 days showed that carprofen + IL-1β had only delayed the GAG release compared with IL-1β (Figure 4).

The degradation of the ECM in articular cartilage is known to be facilitated by pro-inflammatory cytokines such as IL-1β in degenerative joint diseases such as OA [7,8]. Consequently, IL-1β is frequently used in culture models of arthritis to mimic the environment present within OA joints [18,19]. The use of physiologically relevant tissue-culture models (Figure 1) can provide mechanistic insights into the inflammatory and catabolic responses of cartilage by using tissues from euthanized cadavers, reducing the need to use animal models. In this study, we used IL-1β-stimulated cartilage explants in a static culture system. We previously used a targeted high-throughput proteomic approach to identify the major proteins present in the secretome of articular cartilage exposed to IL-1β [10,11].

Here we compared the effects of stimulation with IL-1β in the presence and absence of carprofen. One of the important issues addressed was the potential cytotoxic effects of carprofen on chondrocytes within the cartilage explants, especially because the culture system used was serum free and involved long incubation periods. The cytotoxicity of carprofen at 100 μg/ml was assessed by monitoring β-actin release from the explants by using western blotting. Western blot experiments with antibodies to β-actin were used to demonstrate that chondrocytes within the explants did not undergo cell death and lysis when treated with carprofen.

The data obtained indicated that IL-1β stimulation increases β-actin release, but addition of carprofen alone does not increase cytotoxicity compared with untreated controls (see Additional file 2: Figure S2).

Qualitative MS techniques identified a number of proteins including MMP-1, MMP-3, and MMP-13 in the secretome of IL-1β-stimulated samples (Table 1). These proteins were then selected as markers for studying the effects of carprofen. Treatment with carprofen resulted in a qualitative reduction in the levels of these proteins, even in the continued presence of IL-1β.

This qualitative approach also confirmed that MMP-1 and MMP-13 are found only above the identification threshold in IL-1β-stimulated samples, compared with untreated samples. Quantitative western blotting was used to demonstrate that the released levels of all three MMPs are significantly elevated after IL-1β stimulation. IL-1β signaling initiates the active form of NF-κB, causing transcription of pro-inflammatory and proapoptotic genes, initiating the release of various catabolic enzymes, including aggrecanases, cathepsins, ADAMTS, and MMPs (-1, -3, and -13) [20-22].

MMP-13 is a collagenase and has a major role in type II collagen degradation [23,24]. It also has the ability to degrade other collagens present in cartilage (types VI and IX), aggrecan, and various other ECM proteoglycans and constituents [25]. It has been identified in investigations into OA cartilage as a protein that is differentially upregulated in OA [26,27]. The progression of OA has been shown to be slowed by deletion of the *MMP-13* gene in a MLI-induced OA mouse model [28]. MMP-1 is another member of the collagenase family that is effective at cleaving collagen I, II, and III [29]. The enzyme is able to unwind the triple helix of α chains before it cleaves collagen [29]. A quantitative analysis of chondrocytes has shown that IL-1β stimulation caused a significant increase in MMP-1 secretion [30]. Expression of the *MMP-1* gene in equine articular cartilage has also been shown to be upregulated by IL-1β and LPS intra-articular injections [31]. MMP-3 (stromelysin-1) is known to activate both MMP-1 and MMP-13 by cleavage of their propeptide domains [32], which will lead to increased collagen degradation. In this study, we found that MMP-3 was detectable in the untreated secretome, but levels were significantly higher after IL-1β stimulation. OA cartilage secretome has been show to contain increasingly high levels of MMP-3 [33,34] and is shown to be induced in cartilage and chondrocytes by proinflammatory cytokines [30,35,36].

Measurements of MMP-1, MMP-3, and MMP-13 within *in vitro* models of cartilage inflammation can be used as screening systems for drugs and anti-inflammatory compounds. Interestingly, the reduced levels of these enzymes in the presence of carprofen suggests that this NSAID has the capacity to inhibit MMP release and activation and can be used as a reference drug in studies investigating the effects of compounds with potential anti-inflammatory properties. No previous proteomic studies report the use of carprofen as an inhibitor of MMP release and activity in explant cultures of cartilage stimulated with IL-1β. Therefore, this study expands on previous work in this area by testing the effects of carprofen in a novel context [10]. Carprofen is commonly used as a 24-hour treatment for arthritic dogs and is also available for use in large animals, including horses. The COX-2 enzyme is responsible for inducing metabolism of arachidonic acid, leading to the production of various prostaglandins, including PGE_2_, which contributes to inflammatory signaling in synovial joint arthritis. It would therefore be expected that inhibition of COX-2 by carprofen in this explant model would attenuate the inflammatory effects stimulated by IL-1β stimulation. Significant decreases in the IL-1β-stimulated release of active MMP-1, MMP-3, and MMP-13 were observed in the presence of carprofen.

These findings suggest that as well as alleviating pain, the use of this drug may help protect cartilage against the catabolic effects of proteases and therefore afford a certain level of protection against MMP activation and ECM loss.

The involvement of MMP action in the progression of OA has led to studies on the therapeutic effects of MMP inhibitors. Joint damage, osteochondral angiogenesis, and perceived pain were reduced by treatment with MMP inhibitors in a rat meniscal transection model of OA [37]. Whereas MMPs can contribute to collagen degradation, ADAMTS is believed to be the main enzyme responsible for aggrecan, GAG, and proteoglycan loss [38]. A DMMB assay measuring the GAG release in the cartilage explant culture time course indicated that, although inflammatory MMPs are significantly reduced by carprofen, equivalent GAG release occurs when comparing IL-1β and carprofen + IL-1β-treated explants cultured for up to 12 days.

## Conclusions

In summary, the cartilage explant model used here has allowed us to use MMP-1, -3, and -13 as biomarkers to study the anti-inflammatory and anti-catabolic effects of carprofen. This may represent a useful approach for screening putative anti-inflammatory drugs with added anti-MMP activity *in vitro* and may be used for testing the effects of naturally occurring compounds on MMP expression in cartilage. This model also incorporates important elements of the replacement, refinement, and reduction of research by using animals (3Rs), thus providing an alternative to *in vivo* models of arthritis.

## Abbreviations

ACN: acetonitrile; ADAMTS: a disintegrin and metalloproteinase with thrombospondin motifs; BSA: bovine serum albumin; COX-2: cyclooxygenase-2; DMEM: Dulbecco’s modified Eagle medium; DMMB: dimethylmethylene blue; DMOADs: disease-modifying osteoarthritis drugs; DTT: dithiothreitol; ECM: extracellular matrix; ESI-TRAP: electrospray ionization-TRAP; ETD: electron transfer dissociation; FN-1: fibronectin 1; GAG: glycosaminoglycan; HRP: horseradish peroxidase; IAA: iodoacetamide; IL-17: interleukin-17; IL-1β: interleukin-1 beta; IL-6: interleukin-6; IL-8: interleukin-8; MMP-1: matrixmetalloproteinase-1; MMP-13: matrixmetalloproteinase-13; MMP-3: matrixmetalloproteinase-3; MMPs: matrix metalloproteinases; MS: mass spectrometry; nanoLC-MS/MS: nano liquid chromatography–mass spectrometry/mass spectrometry; NF-κB: nuclear factor kappa B; NSAID: nonsteroidal anti-inflammatory drug; OA: osteoarthritis; PBS: phosphate-buffered saline; PGE2: prostaglandin E_2_; PVDF: polyvinylidene difluoride; RA: rheumatoid arthritis; SDS: sodium dodecylsulfate; SDS-PAGE: sodium dodecylsulfate polyacrylamide gel electrophoresis; TIMPs: tissue inhibitors of MMPs; TNF-α: tumor necrosis factor alpha; TSP-1: thrombospondin 1

## Competing interests

This article was written by the authors within the scope of their academic and research positions. The authors declare that they have no competing interests. None of the authors has any relationship that could be construed as biased or inappropriate. AM is the coordinator of the D-BOARD Consortium funded by European Commission Framework 7 program (EU FP7; HEALTH.2012.2.4.5–2, project number 305815, Novel Diagnostics and Biomarkers for Early Identification of Chronic Inflammatory Joint Diseases).

## Authors’ contributions

All authors have made substantial intellectual contributions to the conception and design of the study, data acquisition, analysis, and interpretation. AW carried out the experimental work and contributed to data collection, interpretation, and analysis. JRS contributed to the qualitative MS data. AM conceived the study design and supervised AW with the collaboration of SL, DA, and PH. All authors contributed to data interpretation and manuscript preparation. All authors approved the final version submitted.

## Authors’ information

http://cordis.europa.eu/projects/home_en.html

http://ec.europa.eu/research/health/medical-research/severe-chronic-diseases/projects/d-board_en.html

## Supplementary Material

Additional file 1: Figure S1 TSP-1 release stimulated by IL-1β is not significantly decreased after carprofen + IL-1β treatment. Western blots for TSP-1 produced a band at about 125 kDa that was detected only in the presence of IL-1β. Cartilage explants were cultured for 6 days with three treatment replicates from the same individual animal (*n* = 3). Explant treatments: untreated control, IL-1β (10 ng/ml), carprofen (100 μg/ml), or carprofen (100 μg/ml) + IL-1β (10 ng/ml). Click here for file

Additional file 2: Figure S2 Cartilage explant cultures treated with IL-1β or carprofen + IL-1β did not show significant differences in β-actin release. (A) Representative western blot confirming the absence of β-actin in explant cultures that were untreated or treated with carprofen alone. IL-1β and carprofen + IL-1β treatments induced β-actin release, as evidenced by detection of an approximate 42 kDa band. (B) Graphic representation of the densitometric analysis of β-actin bands. An unpaired *t*-test was applied to assess statistical significance. ns, no significant difference; +, positive control for β-actin (equine chondrocyte lysate). Cartilage explants were cultured for 6 days with three treatment replicates from two animals (n = 6). Explant treatments: untreated control, IL-1β (10 ng/ml), carprofen (100 μg/ml), or carprofen (100 μg/ml) + IL-1β (10 ng/ml). Click here for file

## References

[B1] Buckwalter JA Mankin HJ Articular cartilage: tissue design and chondrocyte-matrix interactions Instr Course Lect 1998 15 477 486 9571449

[B2] Kuettner KE Biochemistry of articular cartilage in health and disease Clin Biochem 1992 15 155 163 10.1016/0009-9120(92)90224-G 1633629

[B3] Archer CW Francis-West P The chondrocyte Int J Biochem Cell Biol 2003 15 401 404 10.1016/S1357-2725(02)00301-1 12565700

[B4] Urban JP The chondrocyte: a cell under pressure Br J Rheumatol 1994 15 901 908 10.1093/rheumatology/33.10.901 7921748

[B5] Homandberg GA Wen C Hui F Cartilage damaging activities of fibronectin fragments derived from cartilage and synovial fluid Osteoarthritis Cartilage 1998 15 231 244 10.1053/joca.1998.0116 9876392

[B6] Homandberg GA Guo D Ray LM Ding L Mixtures of glucosamine and chondroitin sulfate reverse fibronectin fragment mediated damage to cartilage more effectively than either agent alone Osteoarthritis Cartilage 2006 15 793 806 10.1016/j.joca.2006.02.003 16581272

[B7] Goldring MB Osteoarthritis and cartilage: the role of cytokines Curr Rheumatol Rep 2000 15 459 465 10.1007/s11926-000-0021-y 11123098

[B8] Goldring MB The role of cytokines as inflammatory mediators in osteoarthritis: lessons from animal models Connect Tissue Res 1999 15 1 11 10.3109/03008209909005273 10770646

[B9] Martel-Pelletier J Alaaeddine N Pelletier JP Cytokines and their role in the pathophysiology of osteoarthritis Front Biosci 1999 15 D694 D703 10.2741/Martel 10525480

[B10] Clutterbuck AL Smith JR Allaway D Harris P Liddell S Mobasheri A High throughput proteomic analysis of the secretome in an explant model of articular cartilage inflammation J Proteomics 2011 15 704 715 10.1016/j.jprot.2011.02.017 21354348 PMC3078332

[B11] Williams A Smith JR Allaway D Harris P Liddell S Mobasheri A 451 Strategies for optimising proteomic studies of the cartilage secretome: establishing the time course for protein release and evaluating responses of explant cultures to IL-1β, TNF-α and carprofen Osteoarthr Cartilage 2011 15 S209

[B12] https://www.rimadyl.com/

[B13] http://www.legislation.gov.uk/uksi/1995/731/contents/made

[B14] Farndale RW Sayers CA Barrett AJ A direct spectrophotometric microassay for sulfated glycosaminoglycans in cartilage cultures Connect Tissue Res 1982 15 247 248 10.3109/03008208209160269 6215207

[B15] Troeberg L Nagase H Proteases involved in cartilage matrix degradation in osteoarthritis Biochim Biophys Acta 1824 15 133 145 10.1016/j.bbapap.2011.06.020 PMC3219800 21777704

[B16] Grossetete M Phelps J Arko L Yonas H Rosenberg GA Elevation of matrix metalloproteinases 3 and 9 in cerebrospinal fluid and blood in patients with severe traumatic brain injury Neurosurgery 2009 15 702 708 10.1227/01.NEU.0000351768.11363.48 19834375 PMC2764327

[B17] Woessner JF Jr Matrix metalloproteinases and their inhibitors in connective tissue remodeling FASEB J 1991 15 2145 2154 1850705

[B18] Fernandes JC Martel-Pelletier J Pelletier JP The role of cytokines in osteoarthritis pathophysiology Biorheology 2002 15 237 246 12082286

[B19] Goldring MB Otero M Tsuchimochi K Ijiri K Li Y Defining the roles of inflammatory and anabolic cytokines in cartilage metabolism Ann Rheum Dis 2008 15 iii75 iii82 19022820 10.1136/ard.2008.098764 PMC3939701

[B20] Sutton S Clutterbuck A Harris P Gent T Freeman S Foster N Barrett-Jolley R Mobasheri A The contribution of the synovium, synovial derived inflammatory cytokines and neuropeptides to the pathogenesis of osteoarthritis Vet J 2009 15 10 24 10.1016/j.tvjl.2007.08.013 17911037

[B21] Lorenz H Richter W Osteoarthritis: cellular and molecular changes in degenerating cartilage Prog Histochem Cytochem 2006 15 135 163 10.1016/j.proghi.2006.02.003 16759941

[B22] Martel-Pelletier J Boileau C Pelletier J-P Roughley PJ Cartilage in normal and osteoarthritis conditions Best Prac Res Clin Rheum 2008 15 351 384 10.1016/j.berh.2008.02.001 18455690

[B23] Knauper V Will H Lopez-Otin C Smith B Atkinson SJ Stanton H Hembry RM Murphy G Cellular mechanisms for human procollagenase-3 (MMP-13) activation: evidence that MT1-MMP (MMP-14) and gelatinase a (MMP-2) are able to generate active enzyme J Biol Chem 1996 15 17124 17131 10.1074/jbc.271.29.17124 8663255

[B24] Reboul P Pelletier JP Tardif G Cloutier JM Martel-Pelletier J The new collagenase, collagenase-3, is expressed and synthesized by human chondrocytes but not by synoviocytes: a role in osteoarthritis J Clin Invest 1996 15 2011 2019 10.1172/JCI118636 8621789 PMC507274

[B25] Shiomi T Lemaitre V D'Armiento J Okada Y Matrix metalloproteinases, a disintegrin and metalloproteinases, and a disintegrin and metalloproteinases with thrombospondin motifs in non-neoplastic diseases Pathol Int 2010 15 477 496 10.1111/j.1440-1827.2010.02547.x 20594269 PMC3745773

[B26] Iliopoulos D Malizos KN Oikonomou P Tsezou A Integrative microRNA and proteomic approaches identify novel osteoarthritis genes and their collaborative metabolic and inflammatory networks PLoS One 2008 15 e3740 10.1371/journal.pone.0003740 19011694 PMC2582945

[B27] Bau B Gebhard PM Haag J Knorr T Bartnik E Aigner T Relative messenger RNA expression profiling of collagenases and aggrecanases in human articular chondrocytes in vivo and in vitro Arthritis Rheum 2002 15 2648 2657 10.1002/art.10531 12384923

[B28] Wang M Sampson ER Jin H Li J Ke QH Im HJ Chen D MMP13 is a critical target gene during the progression of osteoarthritis Arthritis Res Ther 2013 15 R5 10.1186/ar4133 23298463 PMC3672752

[B29] Chung L Dinakarpandian D Yoshida N Lauer-Fields JL Fields GB Visse R Nagase H Collagenase unwinds triple-helical collagen prior to peptide bond hydrolysis EMBO J 2004 15 3020 3030 10.1038/sj.emboj.7600318 15257288 PMC514933

[B30] Calamia V Rocha B Mateos J Fernandez-Puente P Ruiz-Romero C Blanco FJ Metabolic labeling of chondrocytes for the quantitative analysis of the interleukin-1-beta-mediated modulation of their intracellular and extracellular proteomes J Proteome Res 2011 15 3701 3711 10.1021/pr200331k 21692455

[B31] Ross TN Kisiday JD Hess T McIlwraith CW Evaluation of the inflammatory response in experimentally induced synovitis in the horse: a comparison of recombinant equine interleukin 1 beta and lipopolysaccharide Osteoarthritis Cartilage 2012 15 1583 1590 10.1016/j.joca.2012.08.008 22917743

[B32] Nagase H Woessner JF Jr Matrix metalloproteinases J Biol Chem 1999 15 21491 21494 10.1074/jbc.274.31.21491 10419448

[B33] Hermansson M Sawaji Y Bolton M Alexander S Wallace A Begum S Wait R Saklatvala J Proteomic analysis of articular cartilage shows increased type II collagen synthesis in osteoarthritis and expression of inhibin betaA (activin A), a regulatory molecule for chondrocytes J Biol Chem 2004 15 43514 43521 10.1074/jbc.M407041200 15292256

[B34] De Ceuninck F Marcheteau E Berger S Caliez A Dumont V Raes M Anract P Leclerc G Boutin JA Ferry G Assessment of some tools for the characterization of the human osteoarthritic cartilage proteome J Biomol Tech 2005 15 256 265 16461950 PMC2291736

[B35] Stevens AL Wishnok JS Chai DH Grodzinsky AJ Tannenbaum SR A sodium dodecyl sulfate-polyacrylamide gel electrophoresis-liquid chromatography tandem mass spectrometry analysis of bovine cartilage tissue response to mechanical compression injury and the inflammatory cytokines tumor necrosis factor alpha and interleukin-1beta Arthritis Rheum 2008 15 489 500 10.1002/art.23120 18240213

[B36] Catterall JB Rowan AD Sarsfield S Saklatvala J Wait R Cawston TE Development of a novel 2D proteomics approach for the identification of proteins secreted by primary chondrocytes after stimulation by IL-1 and oncostatin M Rheumatology (Oxford) 2006 15 1101 1109 10.1093/rheumatology/kel060 16567360

[B37] Mapp PI Walsh DA Bowyer J Maciewicz RA Effects of a metalloproteinase inhibitor on osteochondral angiogenesis, chondropathy and pain behavior in a rat model of osteoarthritis Osteoarthritis Cartilage 2010 15 593 600 10.1016/j.joca.2009.12.006 20067755 PMC2853084

[B38] Sandy JD Verscharen C Analysis of aggrecan in human knee cartilage and synovial fluid indicates that aggrecanase (ADAMTS) activity is responsible for the catabolic turnover and loss of whole aggrecan whereas other protease activity is required for C-terminal processing in vivo Biochem J 2001 15 615 626 10.1042/0264-6021:3580615 11535123 PMC1222096

