# Supplementary figures and images for: Carprofen inhibits the release of matrix metalloproteinases 1, 3, and 13 in the secretome of an explant model of articular cartilage stimulated with interleukin 1β
Source: Arthritis Res Ther. 2013 Dec 30;15(6):R223. doi: 10.1186/ar4424 (PMC3978949; doi:10.1186/ar4424)

## Slide 1
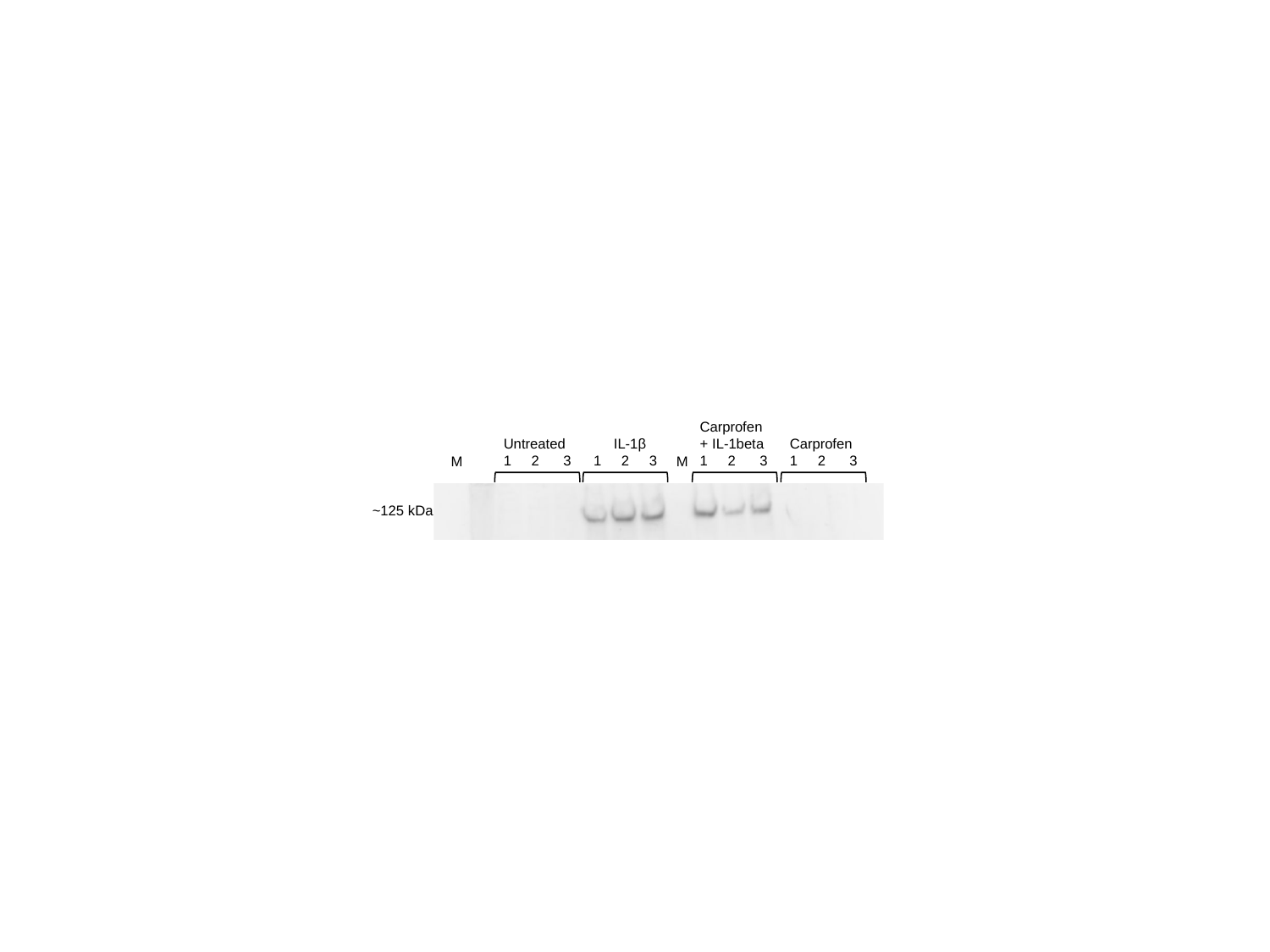

Carprofen
+ IL-1beta
1 2 3
Untreated
1 2 3
 IL-1β
1 2 3
Carprofen
1 2 3
M
M
~125 kDa

Supplement: Additional file 1: Figure S1 — TSP-1 release stimulated by IL-1β is not significantly decreased after carprofen + IL-1β treatment. Western blots for TSP-1 produced a band at about 125 kDa that was detected only in the presence of IL-1β. Cartilage explants were cultured for 6 days with three treatment replicates from the same individual animal (n = 3). Explant treatments: untreated control, IL-1β (10 ng/ml), carprofen (100 μg/ml), or carprofen (100 μg/ml) + IL-1β (10 ng/ml). [file ar4424-S1.ppt]

## Slide 1
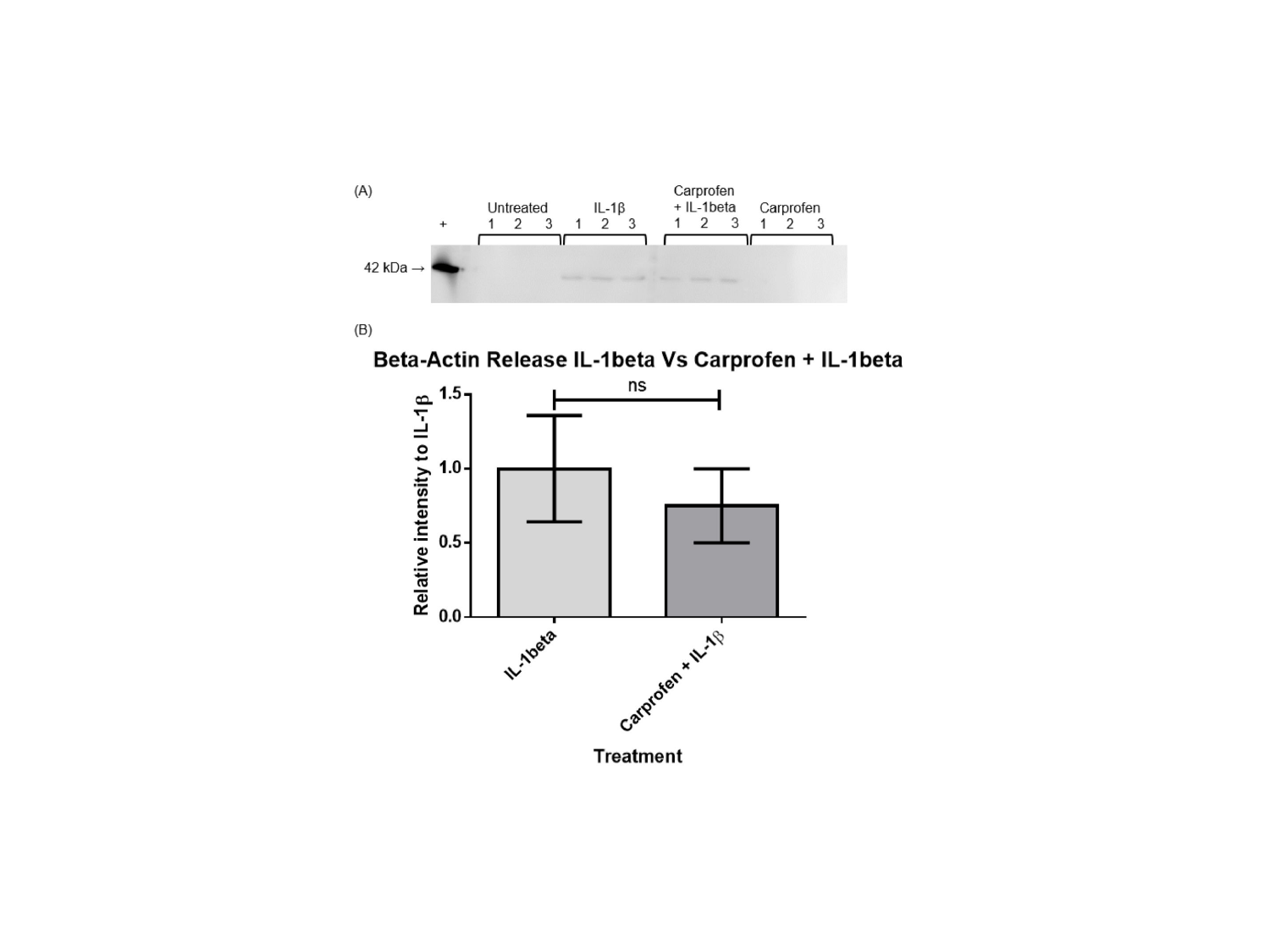

Supplement: Additional file 2: Figure S2 — Cartilage explant cultures treated with IL-1β or carprofen + IL-1β did not show significant differences in β-actin release. (A) Representative western blot confirming the absence of β-actin in explant cultures that were untreated or treated with carprofen alone. IL-1β and carprofen + IL-1β treatments induced β-actin release, as evidenced by detection of an approximate 42 kDa band. (B) Graphic representation of the densitometric analysis of β-actin bands. An unpaired t-test was applied to assess statistical significance. ns, no significant difference; +, positive control for β-actin (equine chondrocyte lysate). Cartilage explants were cultured for 6 days with three treatment replicates from two animals (n = 6). Explant treatments: untreated control, IL-1β (10 ng/ml), carprofen (100 μg/ml), or carprofen (100 μg/ml) + IL-1β (10 ng/ml). [file ar4424-S2.ppt]
